# Half-Bridge Silicon Strain Gauges with Arc-Shaped Piezoresistors

**DOI:** 10.3390/s23208390

**Published:** 2023-10-11

**Authors:** Ji-Hoon Han, Sung Joon Min, Eun-Sang Lee, Joon Hyub Kim, Nam Ki Min

**Affiliations:** 1 KIURI Center for Hydrogen Based Next Generation Mechanical System, Inha University, Incheon 21999, Republic of Korea; 2 Department of Stretchable Task Team, LG Display, Seoul 07796, Republic of Korea; 3 Department of Nanomechatronics Engineering, Pusan National University, Busan 46241, Republic of Korea; 4 Department of Control and Instrumentation Engineering, Korea University, Sejong 30019, Republic of Korea

**Keywords:** strain gauge, bulk micromachining, SiOG MEMS, glass frit bonding, pressure sensor

## Abstract

Half-bridge silicon strain gauges are widely used in the fabrication of diaphragm-type high-pressure sensors, but in some applications, they suffer from low output sensitivity because of mounting position constraints. Through a special design and fabrication approach, a new half-bridge silicon strain gauge comprising one arc gauge responding to tangential strain and another linear gauge measuring radial strain was developed using Silicon-on-Glass (SiOG) substrate technology. The tangential gauge consists of grid patterns, such as the reciprocating arc of silicon piezoresistors on a thin glass substrate. When two half-bridges are connected to form a full bridge with arc-shaped gauges that respond to tangential strain, they have the advantage of providing much higher output sensitivity than a conventional half-bridge. Pressure sensors tested under pressure ranging from 0 to 50 bar at five different temperatures indicate a linear output with a typical sensitivity of approximately 16 mV/V/bar, a maximum zero shift of 0.05% FS, and a span shift of 0.03% FS. The higher output level of pressure sensing gauges will provide greater signal strength, thus maintaining a better signal-to-noise ratio than conventional pressure sensors. The offset and span shift curves are quite linear across the operating temperature range, giving the end user the advantage of using very simple algorithms for temperature compensation of offset and span shift.

## 1. Introduction

Today, high-pressure sensors are widely used in almost all high-tech application areas, from the medical industry, HVAC (heating, ventilation and air conditioning), and general industry to solutions for the transportation industry. High-pressure measurement requires a special sensor design to ensure high performance and safety over a wide temperature range. Commonly used high-pressure sensor technology includes an oil-filled pressure sensor based on the MEMS [1,2,3], a micro fused silicon strain gauge (MSG) sensor [4,5,6,7,8], a thin-film strain gauge pressure sensor [9,10,11] and a thick ceramic film pressure sensor [12,13,14]. The first three key technologies use a steel diaphragm: these stainless-steel transducers can also operate with a full-scale accuracy of 0.5% over a wide temperature range from −40 °C to 150 °C.

**Figure 1:**
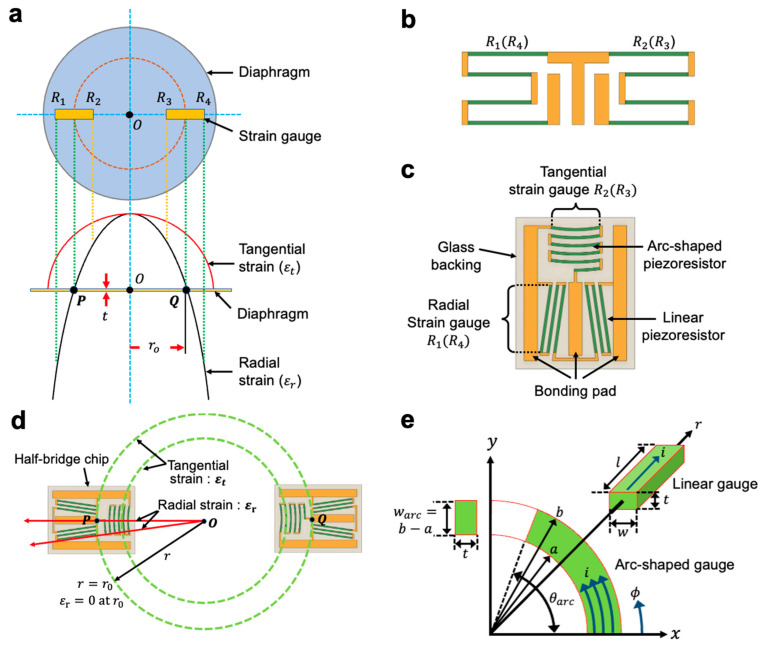
(**a**) Compression and tension distribution on the surface of a stainless-steel diaphragm (*P* and *Q*: the center point where the strain gauge is located above the diaphragm surface), (**b**) structure of a typical half-bridge strain gauge, (**c**) configuration of a half-bridge silicon strain gauge with arc-shaped piezoresistors, (**d**) operation principle of a half-bridge silicon strain gauge chip with arc-shaped piezoresistors and linear piezoresistors, (**e**) design principle of arc-shaped tangential piezoresistor and linear radial piezoresistor on the circular diaphragm.

High-pressure sensors based on strain gauges use a circular metal diaphragm to sense the pressure. The strain distribution in a rigidly clamped circular diaphragm with a uniform pressure distribution is shown in Figure 1a [15,16,17]. With many types of pressure transducers, two gauges are connected close to each other at one or more locations. Figure 1b is a representative example of several configurations that require two gauges at both P and Q locations. In this and similar applications, the two gauges are connected as adjacent bridge arms and act as a half bridge [5,6] with one gauge in tension and the other in compression. This half-bridge strain gauge has several advantages over single gauges: (a) Gauge alignment and bonding are faster and less prone to errors after bonding. (b) As the gauge has a common bond pad, wiring time is reduced. (c) The two gauges are produced from the same local area of silicon wafer and thus have almost identical electrical and thermal properties, which inherently results in a more closely tracked bridge balance as temperature changes [18]. Although the half-bridge gauge in Figure 1b has the various advantages listed above, the full-bridge output consisting of two half-bridges is significantly lower than that of a full-bridge with four single gauges because it only responds to much less radial strain ($\varepsilon_{r}$) in position P(Q) compared with the tangential strain ($\varepsilon_{t})$.

In this paper, we present half-bridge silicon strain gauges with arc-shaped piezoresistors for use on pressure transducers with a metal diaphragm, as shown in Figure 1c. This half-bridge gauge chip is designed to take advantage of the orientation of the tangential and radial strain fields shown in Figure 1a. In addition to the advantages mentioned above, this gauge pattern significantly enhances the full bridge output as the arc-shaped strain gauge responds to tangential strain ($\varepsilon_{t}$) which is much greater than radial strain ($\varepsilon_{r}$), while the conventional half-bridge gauges only measure radial strain. The new half-bridge silicon gauges are fabricated on Si-on-Glass (SiOG) substrates using simple single-sided (top-side) lithography and processing while existing half-bridge gauges are double-sided bulk micromachining. This makes it much easier to fabricate, separate and handle gauge dies compared to conventional gauges [5,19].

## 2. Design of a Half-Bridge Strain Gauge

Figure 1d shows the half-bridge chip composed of a tangential strain gauge and a radial strain gauge and the principle of its operation. This design has both gauges on a common glass substrate and offers advantages such as easier and faster installation and alignment. The tangential gauge consists of six reciprocating arc-shaped piezoresistors that respond to tensile strain. The radial gauge is a meander with six series-connected linear piezoresistors that measure the compressive strain. Two half-bridge gauge chips are symmetrically attached to the diaphragm surface and then connected in a balanced Wheatstone bridge configuration by an aluminum wire bonding to detect small changes in gauge resistance with high accuracy. To achieve the voltage of the differential signal from the applied pressure changes, the half-bridge chips should be located in the region (P and Q in Figure 1d) where the tension and compression gauge areas are close to each other. When pressure is applied to the diaphragm, the tangential strains are always positive, and the radial strains of the opposite sign occur near the edge of the diaphragm. Therefore, the two arc gauges of the bridge measure the positive strain, and the other two meandering gauges measure the negative strain, resulting in a resistance change in each gauge of approximately the same magnitude and opposite sign and a large output voltage.

Figure 1e shows the models for calculating the arc-shaped and linear gauge resistance values. An arc-shaped piezoresistor can be modeled as a resistor with a center angle $\theta_{arc}$ and cross-sectional area ${tw}_{arc}$. The gauge current is in the circumferential direction to measure the tangential component of the strain. The unstressed resistance of this resistor is determined by solving Laplace’s equation ${∆}^{2}=0$, where V is the electric potential, in cylindrical coordinates [20]. Assuming a uniform current and electric field and doping concentration within $\theta_{arc}$, for simplicity, the solution of Laplace’s equation gives the following expression for the arc gauge resistance

(1) $$R_{arc}=R_{S}\frac{\theta_{arc}}{ln(b/a)}$$

where $R_{S}=\rho /t$ is the sheet resistance and *ρ* and *t* are the resistivity and thickness of the silicon layer, respectively.

The arc-shaped piezoresistors can be configured with a different number of turns depending on the desired gauge resistance and sensitivity. For this paper, a five-turn configuration was selected as shown in Figure 1c. The total resistance of the tangential strain gauge is given by the formula

(2) $$R_{tangential}=5.5R_{S}\frac{\theta_{arc}}{ln(b/a)}$$

In Equation (2), factor 5.5 is due to the fact that five and a half (5.5) identical arc piezoresistors are connected to a rotating aluminum piece to obtain the tangential current flow through the strain gauge and minimize the transverse sensitivity of the measuring piezoresistor.

For a linear strain gauge, the current is in the same direction as the piezoresistor orientation as shown in Figure 1e. Therefore, the resistance of an unstressed linear piezoresistor is simply given by

(3) $$R_{linear}=R_{S}\frac{w}{l}$$

where w and l are the width and length of the linear resistor, respectively. Figure 1c illustrates the layout of the radial strain gauge that has six linear piezoresistors connected in series with thin aluminum. The resistance of the radial gauge is given by the formula

(4) $$R_{radial}=6R_{S}\frac{w}{l}$$

As can be seen in Figure 1c, silicon strain gauges typically require multiple turns of the piezoresistor to achieve the desired resistance and higher output voltage. In this case, the two longitudinally oriented piezoresistors are connected by a short transverse loop which can reduce the relative change in resistance. In this paper, as shown in Figure 1c and Table 1, in the proposed Si strain gauge structure, the length (l) of the gauge is overwhelmingly larger than the width (w) (more than 100 times), so the resistance change is dominated by $\pi_{a}$ and $\sigma_{a}$. Therefore, $\pi_{t}$ and $\sigma_{t}$, due to transverse piezoresistive effects, are not considered. The relative resistance change in the structure in Figure 1c can be written as [21]

(5) $$\frac{∆R}{R}=\frac{{∆R}_{l}+{∆R}_{t}}{R_{l}+R_{t}}$$

where $R_{1}$ and $R_{t}$ are the longitudinal and transverse resistance, respectively. From Equation (5) above, the strain gauge should be designed to maximize the ratio of longitudinal to transverse resistance to minimize the effect of the end loops in Figure 1e, all tangential and radial piezoresistors are connected by a turn-around of the aluminum piece, since Al has a much lower resistance and gauge factor than silicon. Table 1 summarizes the geometric parameters of the newly designed half-bridge silicon strain gauge.

## 3. Simulation of Pressure-Induced Strains under Half-Bridge

**Figure 2:**
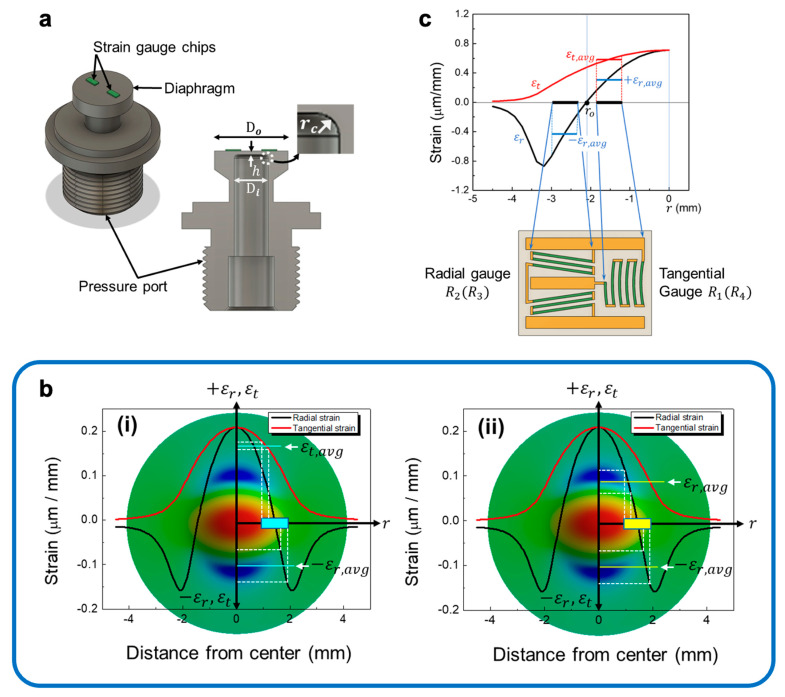
(**a**) Schematic perspective view and a cross-sectional view of a pressure port element with two half-bridge strain gauge chips. (**b**) Results of the finite element analysis of strains of the diaphragm under normal pressure of 50 bar. The curves of the radial change ($\varepsilon_{r}$) and tangential ($\varepsilon_{t}$) strain along the diaphragm radius ($R_{i}$) are superimposed on the strain maps on the diaphragm surface. (**i**) Newly designed gauge: one responds to tangential strain $(\varepsilon_{t})\;$and the other to radial strain ${(\varepsilon }_{r})$, (**ii**) conventional gauges: both gauges respond only to radial strain ($\varepsilon_{r})$. (**c**) Strain distribution and gauge positions on the surface of a stainless-steel circular diaphragm for calculating the average strain.

Figure 2a schematically illustrates a perspective view and a cross-sectional view of a concentric pressure port element with two half-bridge strain gauges. The central axis of the diaphragm coincides with the port central axis.

The output voltage of the strain gauge bridge circuit depends on the average strain under the gauge filaments. The finite-element modeling tool ANSYS was used to verify the design and determine the location of the gauge on the top surface of the circular diaphragm. The strain distribution within the gauges was investigated with the diaphragm being uniformly loaded with pressure. The diaphragm material was 630 stainless steel (SUS 630). The pressure range depends on the parameters of the diaphragm structure such as thickness (h), outer diameter ($D_{o}={2R}_{o}$), inner diameter ($D_{i}={2R}_{i}$) and fillet radius (r). The parameters used in the simulation and calculation are as follows: diaphragm radius R=60 mm, thickness h=4.5 mm and fillet radius r=0.5. The Young’s modulus and Poisson’s ratio of the steel diaphragm were assumed to be $E=1.93\times 10^{11}$ Pa and l=0.31, respectively. The air pressure of 50 bar was evenly distributed across the diaphragm.

Figure 2b shows the strain maps for the tangential and radial directions as well as the detailed strain distribution along one diameter of the diaphragm superimposed on it. As shown in Figure 2b, both tangential and radial strains reach an identical maximum value at the center of the circular diaphragm. The tangential strain is always positive and gradually decreases to zero at the periphery of the diaphragm. The radial strain decreases faster from maximum to zero as the radius increases, becoming negative, and again approaching zero at the edge due to the presence of the fillet radius. It is clearly visible that in the positive (tensile) strain region, the tangential strain is much greater than the radial component, while the radial strain becomes greater in the negative region. Therefore, we designed the reciprocating arc-shaped gauges to respond to tangential strain and the meandering gauges subjected to radial strain, as shown in Figure 1d. The average strains ($\varepsilon_{t,avg\;},\;\varepsilon_{r,avg}$) experienced by each strain gauge were calculated from the two curves in Figure 2b.

## 4. Fabrication of Silicon Gauges

**Figure 3:**
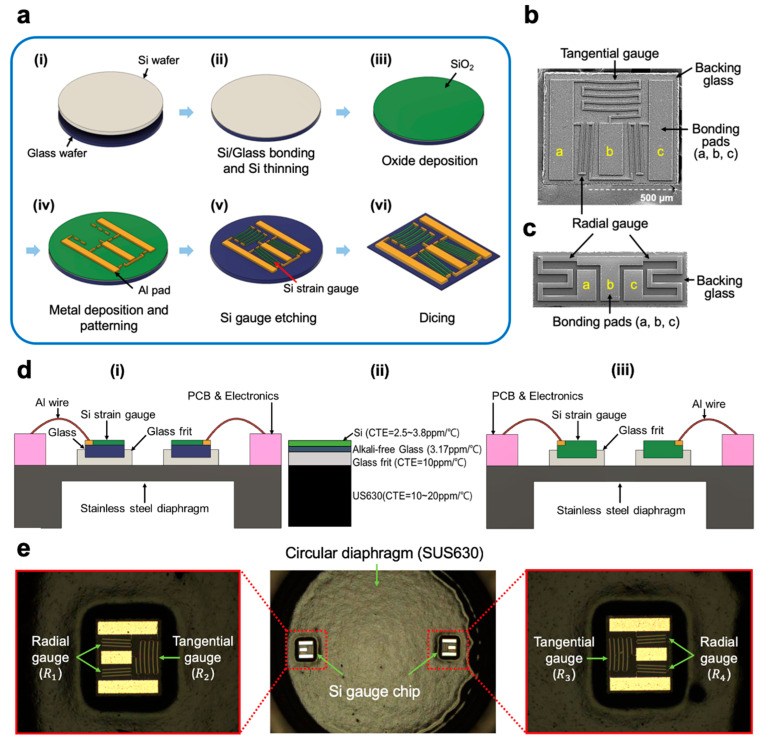
(**a**) Process flow for the production of half-bridge silicon strain gauges: (**i**) the process starts with a low-resistivity Si wafer and an alkali-free glass wafer; (**ii**) silicon/glass wafer anodic bonding and Si thinning; (**iii**) PECVD oxide deposition; (**iv**) metal deposition and patterning by lift-off process; (**v**) Si strain gauge etching by DRIE; (**vi**) dicing. SEM images of fabricated devices: (**b**) the newly designed half-bridge strain gauge chip; (**c**) the conventional half-bridge gauge chip for comparison. (**d**) Half-bridge die and steel diaphragm assembly. (**i**,**ii**) Newly designed sensor: Si/Glass/Glass frit/steel system, (**iii**) conventional sensor: Si/glass frit/steel system, and (**e**) SEM images of two half-bridge strain gauge dies bonded on a glass frit applied to the surface of the thin diaphragm of the pressure port.

The half-bridge silicon strain gauges with arc-shaped piezoresistors were fabricated on an anodically bonded thin silicon-glass wafer using combined bulk micromachining and chemical mechanical polishing (CMP) technology. The single-side fabrication process flow is outlined in Figure 3a. The process started with low resistivity (approximately 0.02 Ω cm or less) of an 8″ p-silicon wafer and an alkali-free glass wafer (EAGLE XG^®^, Corning Inc., Corning, NY, USA) [22], as shown in Figure 3a(i). The silicon resistivity must be sufficiently low since the strain gauges are formed on this wafer without additional impurity dopings. The high-quality glass wafer has a thermal expansion coefficient (CTE) of $3.2\times 10^{-6}$, which is very close to that of silicon, so it can be used as a very thin backing.

A silicon wafer was bonded to a glass wafer with an anodic bonder (model SB6 wafer bonder, Karl Süss, Garching, Germany) (Figure 3a(ii)). The bonding process was carried out at a temperature of 500 °C with a voltage of 1750 V for 40 min in a vacuum [23]. Bonded wafers were evaluated by the naked eye, infrared camera and C-SAM (Scanning Acoustic Microscope, FineSAT III, Hitachi, Japan) [24]. After bonding the Si-glass wafers, the upper silicon was thinned to 10 μm via chemical-mechanical polishing (CMP). The initial oxide layer (Figure 3a(iii)) was then deposited on the thinned silicon surface using plasma-assisted chemical vapor deposition (PECVD) instead of thermal oxidation due to the low-temperature requirement (<600 °C) for wafer glass.

The contact windows were then opened into the oxide using photolithography and reactive ion etching (RIE). A metal stack consisting of 20 nm chromium and 150 nm cobalt titanium was deposited on a patterned resist (suitable for lift-off) via photolithography. After metal deposition, lift-off was performed to produce patterns for the resistor connection and bonding pad (Figure 3a(iv)). Subsequently, the half-bridge silicon strain gauges were defined via optical lithography and then etched using deep reactive ion etching (DRIE) equipment (Multiplex Pro ASE HRM Deep Reactive Ion Etcher, Surface Technology Systems, Newport, UK), as shown in Figure 3a(v). Finally, the rear-side glass was thinned to about 50 μm by chemical–mechanical polishing, and then the entire wafer was diced into strain gauge chips using a saw machine (Figure 3a(vi)). Figure 3b shows one version of the completed half-bridge strain gauge chips. Each chip has two Si gauges and three bond pads. For comparison, a conventional half-bridge silicon gauge was also fabricated according to the same procedure as shown in Figure 3c.

## 5. Gauge Chips and Diaphragm Assembly

A SUS630 stainless-steel diaphragm with glass-bonded half-bridge silicon strain gauges was used to fabricate pressure sensors for evaluation. Bonding the glass frit for silicon strain gauges improves hysteresis and repeatability, increases electrical insulation and widens the operating temperature range compared to conventional organic epoxy adhesives, which exhibit ductile behavior, resulting in hysteresis and zero instability with increasing temperature [25]. Despite these advantages, the most serious problem with this bonding technique is that the difference in the coefficient of thermal expansion (CTE) between the silicon gauge, glass frit and steel diaphragm is large, causing the strain gauge to break. A solution to overcome this problem is shown in Figure 3d(i) compared to a conventional device. In conventional assembly in Figure 3d(iii) [5], the strain gauges are attached directly to the steel diaphragm, and high thermal stresses and cracks occur in the gauge chip due to the CTE mismatch between the steel diaphragm and silicon strain gauge. Therefore, a thin silicon gauge (about 50 μm–100 μm) and a thick glass frit (about 100 Å) were used to relieve the thermal stresses between the steel substrate and the silicon gauges. On the other hand, the arc-shaped gauge assembly system consists of Si/glass/glass frit/steel diaphragm as shown in Figure 3d(ii), and the glass frit thickness can be reduced to 30 μm without die breakage due to the Si-glass system whose CTE matches each other. Moreover, this construction significantly improves the electrical insulation of the device as a layer of good quality glass blocks the leakage current to the diaphragm.

The bonding of the glass frit for the newly developed half-bridge gauge does not differ from that used with conventional silicon strain gauges [7,26,27]. The bonding process was carried out in three main steps: screen printing of the glass paste, its thermal conditioning and actual bonding [26,27,28]. Thermal conditioning transforms the glass paste into a glass layer. The glass frit bonding, starting with the alignment of the strain gauge dies, takes place in the bonding chamber, and the gauge dies are heated to about 430 °C for ten minutes. It is important to prevent voids to form inside the glass frit layer.

Figure 3e shows SEM images of two arc-shaped strain gauge dies bonded to a steel diaphragm using a glass frit. A die shear test was performed on 20 die attachments with a die shear tester (HAWK-8000M, SETEK inc., South Korea) to evaluate the bond strength. The average value of the shear strength for the arc gauge die attachment was equal to 12.8 MPa. All the failures in the die shear testing resulted only from the separation of the silicon gauge die from the glass frit. No separation of the glass frit from the metal diaphragm was observed in any of the 20 die attachment samples. This result would be due to the strong bond between the silica atoms present in the glass frit and the metal atoms on the metal surface through the oxygen atom [29].

## 6. Results and Discussion

The strain gauge chips and diaphragm assembly were attached to the high-pressure test manifold and placed in a temperature-controlled chamber during all tests, which electrical connection provided to the test device. Each strain gauge was tested over five runs under specified conditions of time, temperature and pressure. The resistance changes in the strain gauge were measured with digital multimeters and recorded simultaneously, in real time, using a computer. The bridge excitation was kept constant at 5 V. Before connecting the four strain gauges via a Wheatstone bridge configuration, each individual gauge was evaluated, and then the bridge output of the pressure transducer was examined under different pressures and temperatures.

### 6.1. Determination of Positions and Average Strains for Half-Bridge Gauges

Correct placement of the strain gauges on the diaphragm is essential because the sensor’s output voltage is determined by their position. Figure 2c shows positions of the tangential and radial strain gauges on the diaphragm and method of calculating the average strain they respond to. One method of determining the positions of the strain gauges consists of several tasks: Firstly, a mathematical model of the pressure port element (see Figure 2a) is created for use in the finite element algorithm. Next, the finite element algorithm is used to determine a circle $r_{o}$ (see Figure 1d and Figure 2c) on the sensing side of the circular membrane using the mathematical model of the port element. The center of the half-bridge chip is placed at a determined radius $r_{o}$, which provides a position for the two strain gauges of the half-bridge where the output sensitivity of the full Wheatstone bridge is best. Strain gauges basically measure the average strain under their footprints because they have a finite size [15,30].

In Figure 2c, the arc-shaped gauge measures the average tangential strain $\varepsilon_{t,avg}$, whereas the conventional gauge responds to the average radial strain $\varepsilon_{r,avg}$. For the arc-shaped gauge, the strain along the filament (piezoresistor) is constant, so the average strain is the same as that of the center strain. However, each resistor responds to different average strain because the tangential strain varies along radial direction. Therefore, the average strain over the arc gauge area can be written as [31]

(6) $$\varepsilon_{t,avg}=\frac{\varepsilon_{ab}+\varepsilon_{cd}+\varepsilon_{ef}+\varepsilon_{gh}+\varepsilon_{ij}+\varepsilon_{kl}}{6}\;$$

where 6 is a number of filaments (piezoresistors), and each ε represents an average strain along each filament using the points shown in Figure 2c.

On the other hand, the resistance in radial gauge changes along with the average strain in the radial (longitudinal) direction. When a linear strain is in the radial direction, the average strain of each filament is

(7) $$\varepsilon_{1,2}=\varepsilon_{3,4}=\varepsilon_{5,6}=\varepsilon_{7,8}=\varepsilon_{9,10}=\varepsilon_{11,12}=\varepsilon_{avg}$$

and the average strain under the radial gauge is

(8) $$\varepsilon_{r,avg}=\frac{\varepsilon_{1,2}+\varepsilon_{3,4}+\varepsilon_{5,6}+\varepsilon_{7,8}+\varepsilon_{9,10}+\varepsilon_{11,12}}{6}=\varepsilon_{avg}\;$$

where 6 is a number of filaments in Figure 2c. Table 1 summarizes the average strain for tangential and radial gauges. In this study, in the newly designed half-bridge, the average positive strain at the arc-shaped tangential gauge position is 4.5, which is much larger than the conventional radial gauge with an average value of 3.4, whereas the negative strain values for the dual half-bridges are almost the same. Therefore, the output voltage of the new pressure sensor is expected to be much higher than that of the conventional pressure sensor.

### 6.2. Characterization of Individual Strain Gauge and Pressure Sensors Based on Half-Bridge Arc-Shaped Strain Gauges

The individual sensitivity of all four gauges was evaluated prior to connecting the strain gauges with a Wheatstone bridge configuration. Strain sensitivity is a basic property of the strain-sensitive material used in the strain gauge. For a specific gauge design, the term gauge factor (GF) is used to quantify this sensitivity and is defined as:

(9) $$GF=\frac{∆R/R_{o}}{\varepsilon }\;$$

where *R_o_* is the unstrained resistance, $∆R\;(R-R_{o})$ is the resistance change in the gauge and ε is the applied strain. The gauge factor is a measure of the output since the output voltage of the bridge circuits in which the gauges are used is directly proportional to the gauge factor for a given strain level.

**Figure 4:**
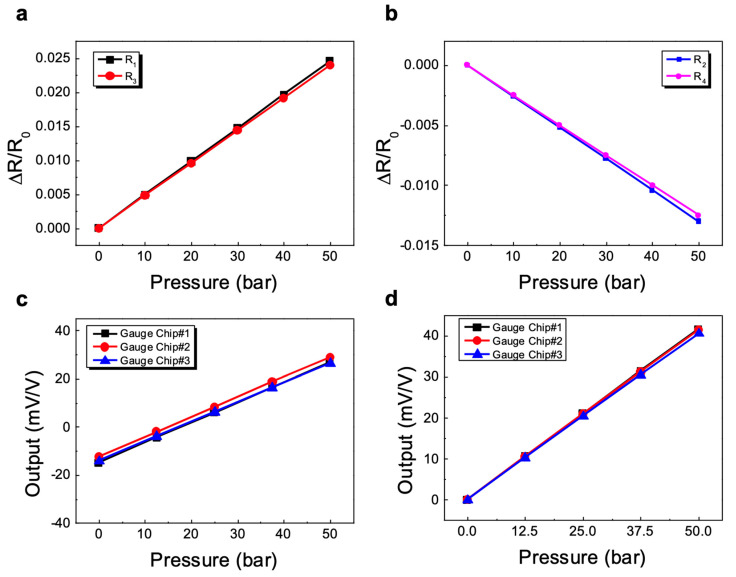
Changes in the relative charge in the gauge resistance with strain for (**a**) two tangential gauges and (**b**) two radial gauges. Variation in the output voltages under pressure for the four basic pressure sensors: (**c**) with an offset shift; (**d**) the bridge offset voltages were eliminated to show the span shift.

Four strain gauges were tested to find the change in resistance as a function of the different strains applied. Figure 4a,b show the change in resistance of individual gauges along with the strain for two tangential gauges ($R_{2}$ and $R_{3}$) and two radial gauges ($R_{1}$ and $R_{4}$) on the edge. It is important to note that the gauge resistance increases with strain for the tangential gauges, and hence, ΔR/R is positive, while the variation ΔR/R is negative for radial gauges located near the edge subjected to compressive stress. It was observed that for all the gauges, the variation in ΔR/R with strain is linear and repeatable (between gauges) over the entire range of strains. This is an excellent performance considering that the gauges were selected randomly from the processed silicon wafer. In the case of two pairs, we can see that both graphs have slightly different slopes. This would be due to the slightly different sensitivity of the strain gauges in production batches, and even for the same wafer, and post-bond misalignment after the bonding procedure was completed.

The gauge sensitivity can be obtained from the slope of the curves using Equation (9). The measured sensitivities and the correlation coefficients from the tested arc-shaped and linear gauges are summarized in Table 2.

To prove their feasibility as a pressure transducer, two half-bridge strain gauges bonded to the diaphragm were connected to a full bridge circuit, the output voltage of these primary pressure sensors was unconditioned and uncompensated, and the performance was tested under the pressure of 0~50 bar.

The variation in the output voltage of basic pressure sensors with a full-bridge strain gauge circuit with applied pressure is shown in Figure 4c. The relationship between bridge output variation and pressure is fairly linear across the pressure range but has different offset voltages. When a bridge-based sensor is installed, the bridge may not output exactly 0 V without load. Slight variations in resistance between the bridge arms generate some non-zero initial offset voltage. To calculate span (sensitivity), the bridge offset was eliminated from Figure 4c, and the net output is plotted in Figure 4d, showing a slight difference in the span between the pressure sensors. The key parameters of the characteristic curve can be found below from the input pressure and output voltage readings. In general, sensor performance is judged by nonlinearity error, repeatability error and hysteresis error [32]. Therefore, during the test, the sensor readings were taken over a minimum of three repeated cycles over a pressure range of 0~50 bar.

The sensitivity S is calculated from the slope of an end-point straight line for each bridge and is usually reported as % of full scale (FS).

(10) S=Span/range

Nonlinearity is the deflection of a characteristic curve or deviation from an ideal straight line. The nonlinearity is calculated by the end-point method. For an output signal indicating the same pressure, this represents the largest difference between the measurements taken in the direction of increasing and (subsequently) decreasing pressure. The effect is minimal and can be neglected in most applications.

The repeatability error is used to quantify the ability of a sensor to continuously deliver the same result under the same circumstances [33]. It can be expressed as the maximum difference between the output readings as determined by the calibration cycles. It is usually presented as % of full scale [34]:

(11) $$\delta_{r}=\frac{∆}{FS}\times 100\%$$

where Δ is the maximum difference between the bridge output readings. The repeatability error was found as d = 0.08% FS of the five runs.

Table 3 compares the results of the performance tests carried out on three arc gauge-based pressure sensors and their conventional analogue. The slight difference in characteristics between the three arc-shaped gauge-based sensors is due to the differences in their dopant concentration, glass frit thickness, post-bonding position and steel diaphragm thickness. The measured sensitivity of the developed pressure sensors has been found to be approximately 1.25 times higher than that of a conventional gauge pressure sensor [4]. This is because arc-shaped gauges respond to much greater tangential strain instead of a lower radial strain, while conventional gauges only measure radial strain. Sensors based on arc gauges, like conventional sensors, demonstrated a high degree of linearity, hysteresis and repeatability. As the table shows, all silicon gauge pressure sensors have a large offset voltage, but the initial value is not an issue unless it is not too large [4,35]. The reason is that the offset is greatly reduced when the pressure sensor is subjected to temperature compensation. In practice, the temperature coefficients (TC) of offset and span shifts are more important than the absolute values, as described below.

### 6.3. Temperature Influence on the Output Characteristics

The change in the ambient temperature results in the corresponding change in three sensor parameters: the offset voltage, the pressure span (sensitivity) and the bridge resistance. The temperature coefficient of resistance (TCR) of a silicon gauge attached to a steel diaphragm is a combination of the TCR of a silicon gauge and the differential thermal expansion between the silicon gauge and the diaphragm. These factors contribute to the gauge’s thermal zero shift or TC offset. The effect of temperature on zero and span is a simple linear approximation of how much the output voltage is altered by changes in temperature. Both are very important for the exact temperature compensation of the pressure sensor. Since many factors will affect offset, each sensor must be temperature tested to determine the sign and magnitude of the compensation.

**Figure 5:**
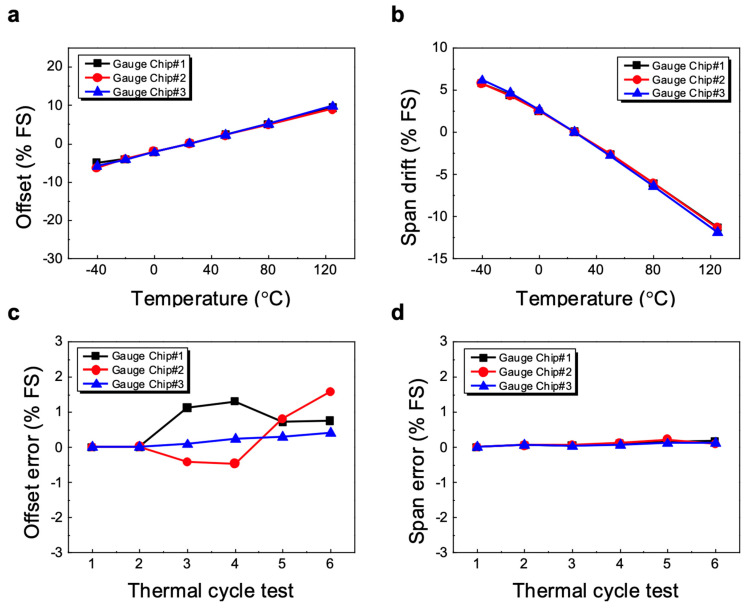
Temperature dependence of (**a**) offset and (**b**) span of basic (uncompensated) pressure sensors. (**c**) Offset and (**d**) span repeatability of basic pressure sensors with the unconditioned and uncompensated output signal (thermal cycle test: 1. Room temperature, 2. High-temperature storage, 3. Low-temperature storage, 4. High-temperature storage (3 days), 5. Low-temperature storage (3 days) and 6. Thermal shock).

Figure 5a,b show the curves for the offset and span shift, respectively. They are fairly linear except for a slight deviation around −20 degrees. The average TC offset value of the three pressure sensors is 0.09% of full scale per 1 °C change, and the TC span is 0.14% FS/°C. This linear relationship in the operating temperature range offers the end-users the advantage of applying a very simple algorithm to compensate for the temperature offset and span shift. Any deviations in the linear function, including those that occur over time and environmental exposures, manifest themselves as sensor errors.

It is important to quantify the offset and span repeatability error for each sensor as this can be used as an early indicator to predict long-term stability and reliability [36,37]. This repeatability refers to the sensor’s ability to reproduce output signal parameters such as offset or span, measured at 25 °C, after exposure to any other pressure and temperature within a specified range. The glass frit bonded sensor assembly undergoes high strain with temperature variations due to the large CTE mismatch between the silicon and the glass frit and steel diaphragm [25,26]. They also suffer from the relaxation process in the glass leading to shift and hysteresis of zero offset at elevated temperature [38]. The stability and hysteresis of the zero offset as well as the sensitivity were tested after different temperature cycles.

Figure 5c,d show the change in the zero offset and the span after several thermal cycles and shock tests, respectively. There was no change in offset after the high-temperature test (125 °C), but the offset for each device that passed the low-temperature test (−40 °C) started to deviate from zero but remained within <2% FS. As shown in Figure 5d, in contrast to the offset, there is hardly any change in the span of the sensor even after each temperature cycle test, which is a characteristic observed in most silicon pressure sensors, because the sensitivity of the strain gauge depends mainly on the impurity concentration in the silicon, not from CTE mismatch. The above experimental results suggest that the long-term stability of a silicon gauge pressure sensor depends mainly on the offset voltage.

## 7. Conclusions

We developed a new Si-on-glass (SOG) half-bridge silicon strain gauge using MEMS processing technology. These are as easy to use, versatile and inexpensive as metal foil gauges, but they have a typical gauge factor close to 100. The developed sensor showed 1.25 times higher output sensitivity compared to the existing silicon half-bridge. This is because arc gauges respond to tangential strain, while conventional half-bridges respond to radial strain. Since the developed silicon gauge is covered with thin glass, it is possible to pick up and place the sensor die from the wafer on the diaphragm surface for an automated bonding process. This significantly reduces labor and increases the yield, enhancing price competitiveness. Moreover, all gauges are fabricated on the front side of the silicon wafer only, making it much more convenient and economical than traditional silicon gauges which require front-to-back alignment followed by deep anisotropic silicon etching on the back side. The high output sensitivity, the easy-to-use gauge is expected to enable a new breakthrough in high-pressure sensor technology. Ongoing research is focused on reducing the offset shift of unconditioned pressure sensors. Preliminary results show that if the glass frit bonding process improves, the offset error after the thermal cycle test can be within 0.5% FS. Another effort is the expansion of the half-bridge technology to full-bridge silicon gauges for pressure or force sensors.

## Figures and Tables

**Table 1:** Summary of geometric dimensions of tangential and radial silicon strain gauges and average strain.

Geometric Dimensions		Average Strain	
Tangential Gauge	Radial Gauge	Tangential Gauge	Radial Gauge
thickness t=10 μm	thickness t=10 μm	$\varepsilon_{a.b}=0.193,\;\;\;\;\varepsilon_{c.d}=$0.191 $\varepsilon_{e.f}=0.192,\;\;\;\varepsilon_{g.h}=0.193$ $\varepsilon_{i.j}=0.191,\;\;\;\;\varepsilon_{k.l}=0.092$	$\varepsilon_{1.2}=\varepsilon_{3.4}=\varepsilon_{5.6}=\varepsilon_{7.8}=\varepsilon_{9.10}=\varepsilon_{11.12}=-0.107$
width $w_{arc}=15\;\mu m$	width w=15 μm		
total length $l_{total}=1650\;\mu m$	total length $l_{total}=1650\;\mu m$	$\varepsilon_{t,avg}=$0.175	$\varepsilon_{r,avg}=$−0.107

**Table 2:** List of sensitivity of tangential and radial gauges according to Equation (9).

Silicon Gauge	Gauge Factor (GF)	Linearity
Radial gauge ($R_{1}$)	119.62	0.99
Tangential gauge ($R_{2}$)	139.71	0.98
Tangential gauge ($R_{3}$)	136.57	0.99
Radial gauge ($R_{4}$)	116.82	0.99

**Table 3:** Comparison of the main characteristics of the two basic pressure sensors based on different half-bridge strain gauges.

Arc Gauge-Based Pressure Sensor	Offset (%FS)	Span (%FS)	Sensitivity (mV/V/bar)	Linearity (%FS)	Repeatability (%FS)	Hysteresis (%FS)
Full-bridge_1	0.05	0.04	0.83	0.06	0.08	0.03
Full-bridge_2	0.04	0.03	0.83	0.08	0.08	0.04
Full-bridge_3	0.05	0.03	0.81	0.07	0.08	0.02
Conventional gauge-based pressure sensor [4]	0.05	0.03	0.66	0.07	0.10	0.03

## Data Availability

The data presented in this study are available on request from the corresponding author.
